# First reported congenital lumbar hernia with lumbo-costo-vertebral syndrome in the Middle-East: a case report

**DOI:** 10.1093/jscr/rjab254

**Published:** 2021-06-22

**Authors:** Aghyad Kudra Danial, Mohamad Morjan, Ahmadfateh Assi, Hasan Raslan, Mario Bedon, Hayat Khalil, Nour Kalaji

**Affiliations:** Department of Surgery, Aleppo University Hospital, Aleppo 15310, Syria; Department of Pediatric Surgery, Aleppo University Hospital, Aleppo 15310, Syria; Department of Pediatric Surgery, Aleppo University Hospital, Aleppo 15310, Syria; Faculty of Medicine, University of Aleppo, Aleppo 15310, Syria; Faculty of Medicine, University of Aleppo, Aleppo 15310, Syria; Faculty of Medicine, University of Aleppo, Aleppo 15310, Syria; Faculty of Medicine, University of Aleppo, Aleppo 15310, Syria

## Abstract

Congenital lumbar hernia is a rare disease affecting mainly infants. Its association with lumbo-costo-vertebral syndrome (LCVS) is hardly seen in the literature. We present a case of LCVS in a 1-month-old female infant presenting with a soft bulge in left lumbar region. Abdominal X-ray revealed absence of multiple ribs with a mild lumbar scoliosis and defective ninth vertebra. Ultrasonography showed absence lateral abdominal wall muscles in left lumbar region and 2.5 cm diameter lumbar hernia. Primary repair of the adnominal wall defect was performed without meshplasty and no recurrence was reported. We hope to enhance the literature of this rare disease with our case.

## INTRODUCTION

Congenital lumbar hernia (CLH) is a rare entity, which is scarcely reported in the English literature [1]. The lumbar hernia classically occurs in the superior lumbar triangle (Grynfeltt–Lesshaft triangle), the inferior lumbar triangle (Petit triangle), diffusely or outside of those triangles in the lumbar area [2]. Lumbar hernia was first suggested by Barbette in 1672 [3]. It can present by itself or associated with various defects, most commonly with lumbo-costo-vertebral syndrome (LCVS) [4], making the combination of both CLH and LCVS an extremely unusual presentation. It may be present at birth or noticed in older age groups [5]. We present to you the first reported case of CLH associated with LCVS in the Middle East in a 1-month-old female infant.

## CASE PRESENTATION

**Figure 1:**
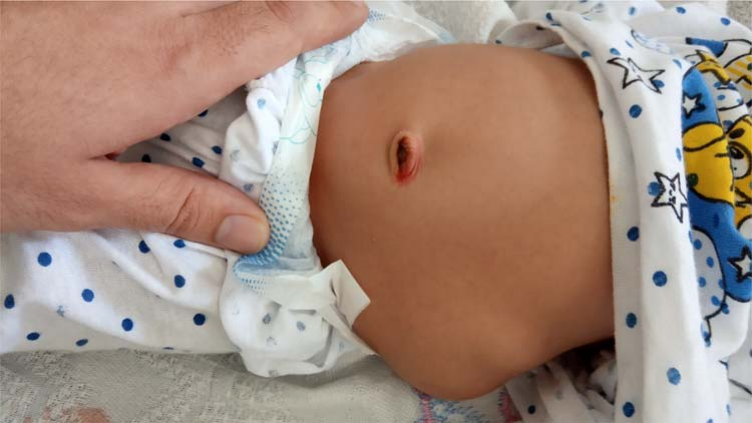
The CLH presenting as a bulge in the abdomen.

A 1-month-old girl presented to the surgery department with a left-sided lumbar bulge (Fig. 1), which was present since birth. It became prominent on crying. The perinatal history is normal, and she weighed 2.4 kg at birth.

Physical examination revealed a soft mass in the left lumbar region with no abnormalities on overlying skin and no splanchnomegaly. She weighed 3.2 kg at presentation. Examinations of her reflexes, cardiac system and anus were all normal.

**Figure 2:**
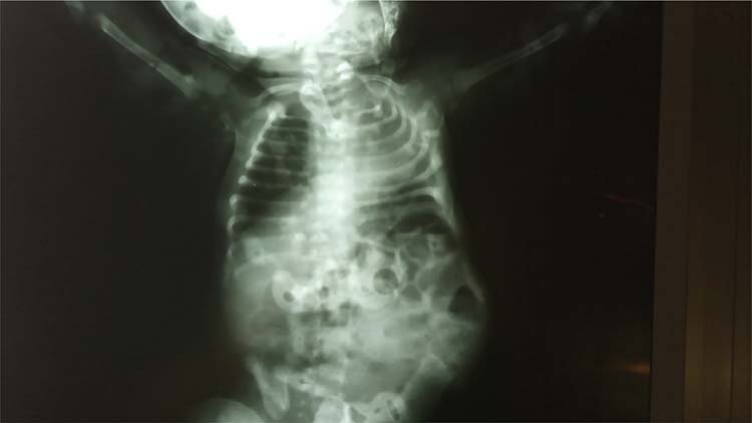
X-ray film shows absence of ribs and mild lumbar scoliosis.

**Figure 3:**
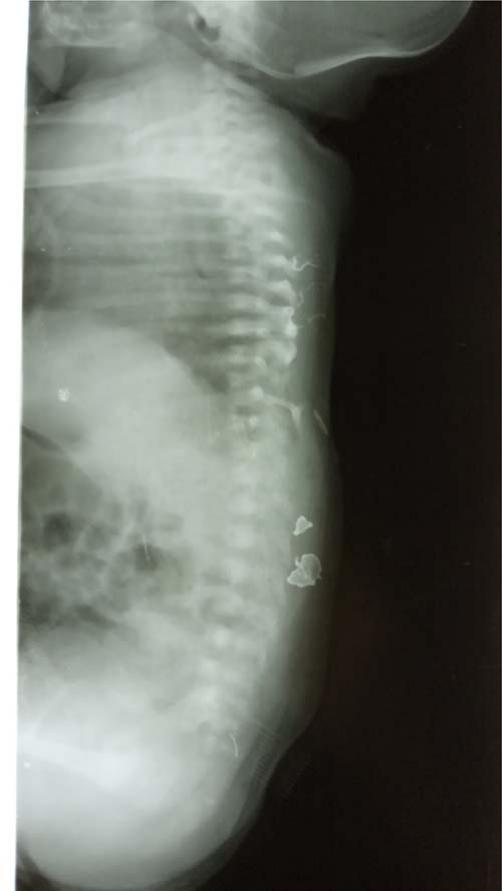
Lateral abdominal x-ray showing the defected vertebra.

Abdominal X-ray revealed absence of the left 10th to 12th ribs along with half of the 9th rib and a mild lumbar scoliosis (Fig. 2). The shadow of the intestines was seen close to the left abdominal wall. Lateral abdominal X-ray showed a defective ninth hemivertebra (Fig. 3). Abdominal sonography showed absence of muscles of the lateral abdominal wall in the left lumbar region and a lumbar hernia with a diameter of 2.5 cm. Liver, spleen and both kidneys were normal. Laboratorial tests did not raise any suspicions.

**Figure 4:**
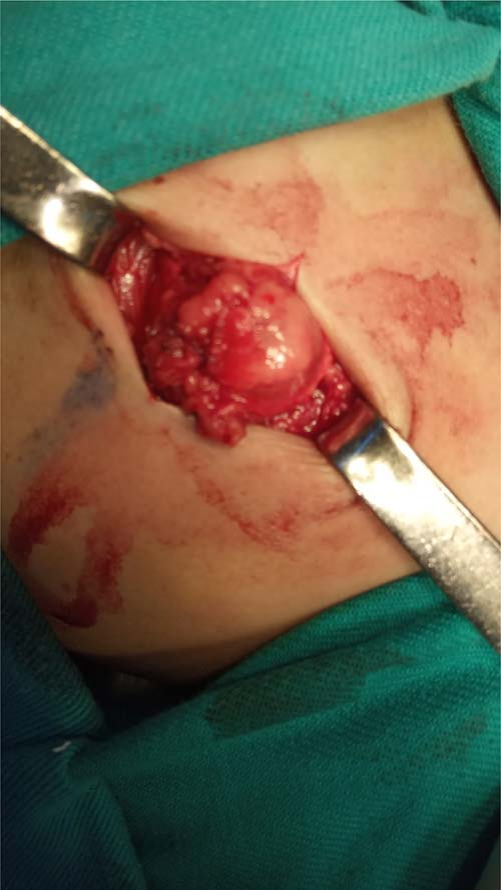
Dissection stage of the hernial sac.

**Figure 5:**
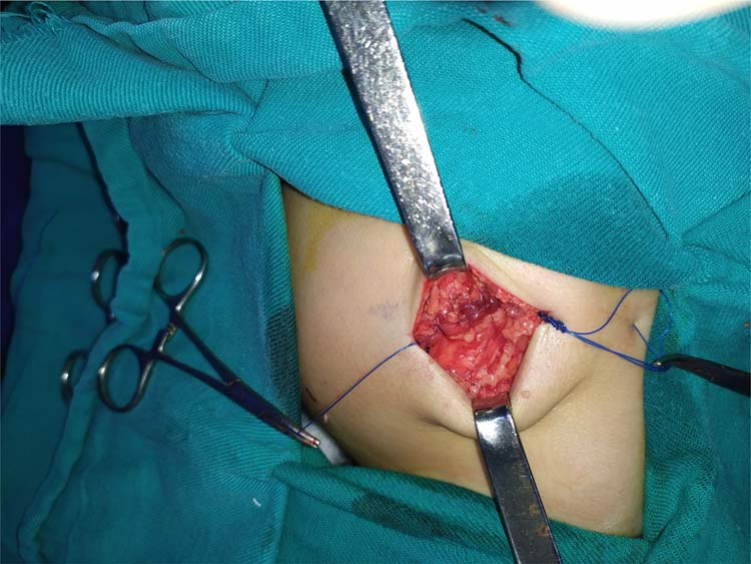
The consolidation stage after reduction of the hernia sac of the abdominal cavity.

After studying the case, we decided to perform primary repair of the abdominal wall defect without meshplasty since the diameter of the aperture was <5 cm. An oblique left lumbar incision was performed above the hernia, revealing a defect measuring 4 cm in diameter in the upper lumbar triangle. Dissection of the peritoneum and hernial sac was performed starting from the margins of abdominal muscles (Fig. 4), the hernial sac was internally reduced and the muscular margins were closed by simple continuous sutures made from prolene 0 (Fig. 5). Complete closure of the defect was confirmed and a draining tube was inserted subcutaneously before closing the skin. The drain was removed after 2 days; the stitches were removed after 10 days. The patient was faring well after the surgery. Recurrence of the hernia was not reported. We recommended that the patient should be followed up by an orthopedist for the correction of disability, which is caused by LCVS.

## DISCUSSION

CLH is one of the rarest varieties of abdominal parietal hernias. In 1672, Barbette was the first to suggest the existence of lumbar hernia, whereas the first case of CLH was reported by Garangeot in 1731 [3]. Lumbar hernias can be divided congenital and acquired (spontaneous, traumatic and iatrogenic), and CLH is considered the most rare [1]. The superior lumbar (Grynfeltt–Lesshaft) triangle is surrounded by the following entities: superiorly by the 12th rib, laterally by the internal abdominal oblique muscle and medially by the quadratus lumborum muscle. Its floor is the transversalis fascia and its roof is the external oblique muscle. The inferior (Petit) lumbar triangle is surrounded by the iliac crest inferiorly, by latissimus dorsi posteriorly and by external abdominal oblique anteriorly. Its floor is the internal oblique muscle. Lumbar hernias occur most commonly in those two lumbar triangles, but they can be diffuse or arise outside of those two triangles above the level of the 12th rib as reported by Akçora *et al*. [2]. LCVS is the most common congenital anomaly seen with CLH [4], but still the combination of CLH and LCVS is rarely reported in the literature. LCVS includes one or more of the following anomalies: hemivertebrae, rib abnormalities, aplasia of dorsolumbar muscles and scoliosis. The convex curvature of the spine eventually faces the hernia.

According to a case series done by Sharma *et al*. [5], the median age at presentation for CLH was 3 months with insignificant gender differences. Out of 18 patients, 12 had the hernia in the inferior lumbar triangle. The hernias occurred equally on both sides. Another study done by Rattan *et al*. [4] showed opposite results. Male-to-female ratio was 6:1 and 12 patients out of 14 had hernias in the superior lumbar triangle. Right-sided hernias were predominant. Bilateral hernia was the rarest in both studies. Therefore, inconsistency exists in the literature of LCVS-associated CLH that is due to the lack of published papers on this extremely rare disease.

Lesshaft found that the superior lumbar triangle developed the hernia more commonly than the inferior lumbar triangle, since the superior lumbar triangle has congenital weak points in its floor, where the 12th dorsal neurovascular bundle perforates the transversalis fascia [6]. But the pathogenesis suggested by Touloukian [7] was attributed to a somatic defect at the third to fifth week of human embryogenesis as a result of transient anoxia. This explains why the defect in the inferior triangle was predominant with Rattan and colleagues. Maternal diabetes has been suggested as a cause of CLH in the offspring [8].

The hernia may be apparent in the newborn, but it typically presents in an older infant as a lumbar mass, which worsens upon crying. The hernial content consists most commonly of intestines, but the spleen or kidney can be found rarely [9]. Computed tomography and ultrasonography are the most useful diagnostic tools to detect hernias and their contents. Surgery is recommended as management, although laparoscopy plays a role nowadays [10]. The size of hernia is the determining factor in management. For a defect of <5 cm, primary repair is done. For a defect of >5 cm, meshplasty should be considered.

In order to create a consistent literature on a specific rare disease, it must be sufficiently equipped with enough cases so that it can give realistic answers and insights and prevent unnecessary doubts and misunderstandings.
